# Determination of Mifepristone (RU-486) and Its Metabolites in Maternal Blood Sample after Pharmacological Abortion

**DOI:** 10.3390/molecules27217605

**Published:** 2022-11-06

**Authors:** Paweł Szpot, Olga Wachełko, Tomasz Jurek, Marcin Zawadzki

**Affiliations:** 1 Department of Forensic Medicine, Wroclaw Medical University, 4 J. Mikulicza-Radeckiego Street, 50345 Wroclaw, Poland; 2 Institute of Toxicology Research, 45 Kasztanowa Street, 55093 Borowa, Poland

**Keywords:** mifepristone, RU-486 metabolism, pharmacological abortion, miscarriage, UHPLC-QqQ-MS/MS, forensic toxicological investigations

## Abstract

The aim of the study was the development and validation of the UHPLC-QqQ-MS/MS method for the determination of mifepristone in human blood as well as the identification and quantification of its metabolites after self-induced pharmacological abortion. The metabolic pathway in humans was proposed after examination of an authentic casework. The fast and simple preanalytical procedure was successfully applied (pH9, *tert*-butyl-methyl ether). The validation parameters of the method were as follows: limit of quantification: 0.5 ng/mL; coefficients of determination: >0.999 (*R*^2^), intra- and inter-day accuracy and precision values did not exceed ± 13.2%. The recovery and matrix effect were in the range of 96.3–114.7% and from −3.0 to 14.7%, respectively. Toxicological analysis of the mother’s blood (collected the day after the pregnancy termination) revealed the presence of five compounds: mifepristone (557.4 ng/mL), *N*-desmethyl-mifepristone (638.7 ng/mL), 22-OH-mifepristone (176.9 ng/mL), *N,N*-didesmethyl-mifepristone (144.5 ng/mL) and *N*-desmethyl-hydroxy-mifepristone (qualitatively). To our knowledge, the study presented in this paper is the first report on the concentrations of mifepristone and its metabolites in maternal blood samples after performing a self-induced abortion. The established UHPLC-QqQ-MS/MS method is suitable for forensic toxicological analysis as well as in terms of clinical toxicology in future investigations (examination of pharmacokinetics, bioavailability and metabolism of RU-486).

## 1. Introduction

Mifepristone (RU-486) has been known as a competitive inhibitor of progesterone since it was first synthesized and widely distributed in the 1980s [1]. Due to its properties, mifepristone is commonly used for pharmacological abortions, which are an alternative to surgical abortions via vacuum aspiration or dilatation and curettage. Mifepristone is usually administered alone or in combination with Rivanol [2] or prostaglandin analogs [3]. Globally, out of 55.7 million abortions that occurred each year between 2010 and 2014, 25.1 million were unsafe. Furthermore, as many as 97% of these unsafe pregnancy terminations (24.3 million) took place in developing countries. Statistically, developing countries have a significantly higher rate of unsafe abortions than developed ones [4]. In turn, there was a significant increase in the number of induced abortions worldwide between 2015 and 2019 (an average of 73.3 million per year). Taking the reproductive age of women (15–49 years old) it was 39 pregnancy terminations per 1000 women, while six out of 10 (61%) of all unintended pregnancies ended in an induced abortion [5]. The issue of abortions is also related to women’s safety and health. An estimated 4.7─13.2% of all maternal deaths are the consequence of an unsafe abortion [6]. In developing countries, in 2012 alone, 7 million women were treated for complications after unsafe pregnancy termination [7]. The costs to health care systems in those countries associated with abortion-related female morbidity and mortality range from $375 million to as much as $838 million [8]. Considering the abovementioned data, the worldwide problem of unsafe abortion seems to be extremely important.

One of the most hazardous methods of terminating a pregnancy is the use of abortion pills of an unknown origin (bought on the internet) by a pregnant woman and inducing the abortion by herself at home without medical supervision or specialist care. The consequences of such a practice can be fatal. As the number of illegal abortions increases, counterfeit medication abortifacients are becoming more widely available on the black market. Considering that the concentration of active ingredients can vary dramatically between different fake abortion-inducing drugs, their illegal distribution can pose a significant public health problem [9]. Mifepristone (one of the active ingredients of abortion pills) is used to induce an abortion during an unwanted pregnancy, and is illegal in some countries. This aspect is particularly important in forensic toxicology. In some countries, mifepristone cannot be legally distributed; therefore, analysis of the blood of women who have had a miscarriage (and/or fetal blood) is necessary to confirm or exclude the possibility of using this drug.

The analytical techniques for mifepristone quantification described to date were only applied for plasma and serum samples examination. Determination of mifepristone was possible by using a high-performance liquid chromatography (HPLC), most commonly with UV detection [10,11,12,13]; only two papers concern the use of more specific detectors such as mass spectrometry (MS) with triple quadrupole (QqQ) [14] or with quadrupole time-of-flight (QTOF) [15].

The aim of this presented study was to develop and validate an ultra-high-performance liquid chromatography coupled with a triple quadrupole mass spectrometry (UHPLC-QqQ-MS/MS) method for the determination of mifepristone and its metabolites in a small volume of a whole blood sample. The developed method was applied for the quantification of mifepristone in a maternal blood sample. The woman was suspected of carrying out a self-induced abortion with the use of pills purchased on the internet. According to our knowledge, this is the first casework to date with the full description of maternal blood concentrations of mifepristone and its metabolites, the time of ingestion and dosage of the drug, as well as symptoms after taking this abortifacient. The [resented casework can provide valuable information for further forensic and clinical investigations. Additionally, in this study metabolites of mifepristone were identified and quantified in the tested blood sample and a potential metabolic pathway of mifepristone in humans was proposed. To our knowledge, the presented study is the first to report blood concentrations of mifepristone and its metabolites in a maternal blood sample after a self-induced abortion.

### Case History

A 22-year-old woman (180 cm, 70 kg) arrived at the hospital with a dead fetus, stating that she had had a miscarriage. The woman was communicative, but appeared to be in shock. She claimed that she had not been aware she was pregnant and had not experienced any symptoms of pregnancy. The woman had no visible injuries. During a search of her apartment, the following drugs were found: five tablets of Seractil 400 mg (dexibuprofen), five tablets of Ospen 1500 mg (phenoxymethylpenicillin), an empty package of A-Kare tablets (mifepristone 200 mg + misoprostol 200 μg), a package containing two tablets called Miso-Gyn (misoprostol 200 μg), and a plastic bag with the contents of a used pregnancy test with two purple lines. During interrogation, she admitted to taking abortion pills; she had found information about their usage on the internet and was expected to pay €80 for them. On the day of the incident, she took four A-Kare pills at 9:00 a.m. and two further pills at 1:00 p.m. (according to the instructions provided with the package). After taking the medicines she had strong diarrhea, and at approximately 8:00 p.m. experienced severe abdominal pain. She testified that at about 8:45 p.m. she stillbirthed a dead fetus. The woman’s blood was collected for testing in the hospital at 9:40 a.m. (the day after the miscarriage). The results of the autopsy on the fetus were as follows: physique appropriate to fetal age. Weight: 286 g, length: 26 cm, gender: female. The dimensions indicated 20–21 weeks of gestation. The autopsy revealed that the fetal cardiovascular function was intact during the labor, and therefore the fetus appeared to be alive at that time. The results of a histopathological examination indicated a three-vessel umbilical cord with a normal structure. The structure of the placenta corresponded to the second or third trimester of pregnancy. Extensive hemorrhages were found within the placenta, possibly indicating placental abruption.

## 2. Materials and Methods

### 2.1. Chemicals and Reagents

Water, acetonitrile and methanol (Chromasolv^®^ LC–MS), ethyl acetate, *n*-hexane, dichloromethane, *tert*-butyl-methyl ether and formic acid were purchased from Sigma-Aldrich (Steinheim, Germany); ammonium formate was purchased from Sigma-Aldrich (Mumbai, India); ammonium carbonate was purchased from Merck (Darmstadt, Germany); mifepristone, mifepristone-*d_3_*, 22-OH-mifepristone, 22-OH-mifepristone-*d_6_*, *N*-desmethyl-mifepristone and *N,N*-didesmethyl-mifepristone were purchased from Toronto Research Chemicals (Toronto, Canada). *N*-desmethyl-mifepristone-*d_3_* was purchased from TLC Pharmaceutical standards (Ontario, Canada). All standards were in the form of neat powders and were dissolved in methanol. The stock standard solutions of four substances were mixed to obtain working solutions at concentrations of 10 µg/mL. Three internal standards were mixed to the final concentration of 1 µg/mL. The standard solutions were stored at a temperature of −20 °C.

### 2.2. Blank Material

Blank samples of human blood were collected during autopsies performed in the Department of Forensic Medicine. Blank samples were screened prior to spiking to ensure that they were free from mifepristone and its metabolites. Blood blank samples as well as maternal blood samples were collected in test tubes with sodium fluoride.

### 2.3. Working Solutions, Quiality Control Samples, Calibration Curve

The working standard solution was diluted with methanol to achieve the following concentrations of mifepristone and its metabolites: 5, 10, 50, 100, 500, 1000, 5000 and 10,000 ng/mL. The working solutions were mixed with human blood to obtain final concentrations of: 0.5 (lower limit of quantification (LLOQ), 1, 5, 10, 50, 100, 500 and 1000 (upper limit of quantification (ULOQ) ng/mL. Quality control samples (QC) were prepared in concentrations of 5 (low QC), 100 (medium QC) and 500 (high QC) ng/mL for mifepristone and its metabolites in human whole blood samples.

### 2.4. Instrumentation

An ultra-high performance liquid chromatograph (UHPLC Shimadzu Nexera X2, Kyoto, Japan) was utilized. The separation was achieved by the use of a Kinetex XB-C18 2.1 × 150 mm × 2.6 μm (Phenomenex, Torrance, CA, USA) column. The thermostat temperature was set at 40 ˚C. A mixture of 10 mM ammonium formate with 0.1% formic acid in water (A) and 0.1% formic acid in acetonitrile (B) was used as a mobile phase. The gradient elution was carried out at a constant flow 0.4 mL/min. The gradient applied was as follows: 0 min—5% B, 12 min—98% B, 14 min—98% B, 15 min—5% B. A return to the started gradient compositions (95% A and 5% B) was performed for 5 min. The injection volume was 2 μL.

Detection of the mifepristone and its metabolites was achieved using a triple-quadrupole mass spectrometer (QqQ, Shimadzu 8050, Kyoto, Japan) with an electrospray ion source (ESI) in positive ionization (+). Determination of substances was carried out in the multiple reaction monitoring (MRM) mode. The following MS parameters were fixed: nebulizing gas flow: 3 L/min; heating gas slow: 10 L/min; interface temperature: 250 °C; desolvation line temperature: 200 °C; heat block temperature: 350 °C; drying gas flow: 10 L/min. A summary of MRM conditions applied in the UHPLC-ESI-QqQ-MS/MS method is presented in Table 1.

### 2.5. Sample Preparation

A volume of 200 µL of a human whole blood sample was transferred to a 12-mL plastic tube. A volume of 20 μL of the internal standard MIX solution (mifepristone-*d_3_*, 22-OH-mifepristone-*d_6_*, *N*-desmethyl-mifepristone-*d_3_*, each substance at a concentration of 1000 ng/mL) and 200 μL of 0.5 M ammonium carbonate solution (pH 9) were added. Liquid−liquid extraction (LLE) with 2 mL of *tert*-butyl-methyl ether was carried out for 10 min. Samples were centrifuged at 1520× *g* (10 min; 4 °C); the organic phase was then transferred to a 2-mL Eppendorf tube and evaporated to dryness under a stream of nitrogen (at 40 °C). The extract was dissolved in 50 μL of methanol, transferred to the glass insert and analyzed by UHPLC-QqQ-MS/MS.

### 2.6. Optimization of Extraction Technique

In order to choose the most suitable extraction technique, different pH conditions (pH 7.4 and pH 9) as well as various organic solvents with different polarity (ethyl acetate, *n*-hexane, dichloromethane, *tert*-butyl-methyl ether) were tested. Two sets of samples (both at a final concentration of 50 ng/mL for each compound): blood samples (*n* = 3) and without a biological matrix (*n* = 3) were prepared. Blood samples were subjected to an extraction procedure under the abovementioned conditions. Samples without the matrix were evaporated to dryness without performing extraction of the analytes. In each set, the peak areas of substances of interest were averaged. Extraction efficiency [%] was determined by comparing the peak area of each substance after extraction (percentage of extraction) to the peak area obtained by evaporation of a stock solution (considered to be 100% extraction efficiency).

### 2.7. Identification of Metabolites

The first step in the identification of mifepristone metabolites was analysis of the mother’s blood extract using the ultra-high performance liquid chromatography tandem triple quadrupole mass spectrometry (UHPLC-QqQ-MS) in the Q3 scan mode. The parameters of the chromatographic separation and mass spectrometer conditions were identical as described above (Section 2 paragraph). The second step was an analysis performed in the product ion scan mode (at three different collision energies, −10, −20 and −35 V) for the mifepristone and all identified metabolites of this compound. After identification of metabolites, a proposed metabolism pathway was created, and a quantitative method was established in the MRM mode.

### 2.8. Method Validation

Evaluated parameters of the method included: selectivity, calibration model, precision and accuracy, carryover, the limit of quantification (LOQ), recovery and matrix effect. Selectivity of the method was established by analyzing five different lots of blank blood for possible endogenous interference peaks at the retention time of the mifepristone and metabolites (*N*-desmethyl-mifepristone, *N*,*N*-didesmethyl-mifepristone, 22-OH-mifeprisone). Linearity was evaluated by an analysis of working solutions human blood in final concentrations of 0.5, 1, 5, 10, 50, 100, 500 and 1000 ng/mL. A linear calibration model was applied and the coefficient of determination (*R*^2^) was determined for each compound. The precision and accuracy were estimated by replicating the analysis (*n* = 5) of QC samples at three concentration levels: 5, 100 and 500 ng/mL of determined compounds. Precision was defined as a relative standard deviation (RSD%), while accuracy was expressed as a mean relative error (RE%). To investigate the carryover, three samples without analytes were analyzed after a calibration sample with the highest mifepristone and its metabolites concentration. Unacceptable carryover was when a peak area ratio in a zero sample after analysis of a sample containing a high concentration of determined compounds exceeded 20% of the area ratio observed for the LOQ samples. The limit of quantification (LOQ) was defined as the concentration at which the relative standard deviation (RSD%) did not exceed 20% and the signal to noise ratio met the condition at least: S/N ≥ 10. The recovery (*n* = 5) was evaluated at each of three concentration levels: low, medium and high QC. The recovery (in percentage) was determined by comparing the response of extracted analytes in spiked blank biological specimens with the response of extracted analytes from samples without a biological matrix. The matrix effect (in percentage) was calculated using an equation described by Chambers et al. [16]. A short-term stability examination was performed by analysis of QC samples (5, 100 and 500 ng/mL) immediately after preparation and, later, after 48 h. The samples were stored in an autosampler at a stable temperature of 5 °C. The substances were considered to be stable when the bias values were not greater than 15%.

## 3. Results and Discussion

### 3.1. Method Development and Validation Results

The results of an extraction procedure optimization are summarized in Table 2. In the case of all substances, pH 9 was the best environment for extraction and *tert*-butyl-methyl ether was the most suitable organic solvent. Both optimized parameters allowed for the best recovery rates of all analytes.

**Figure 1:**
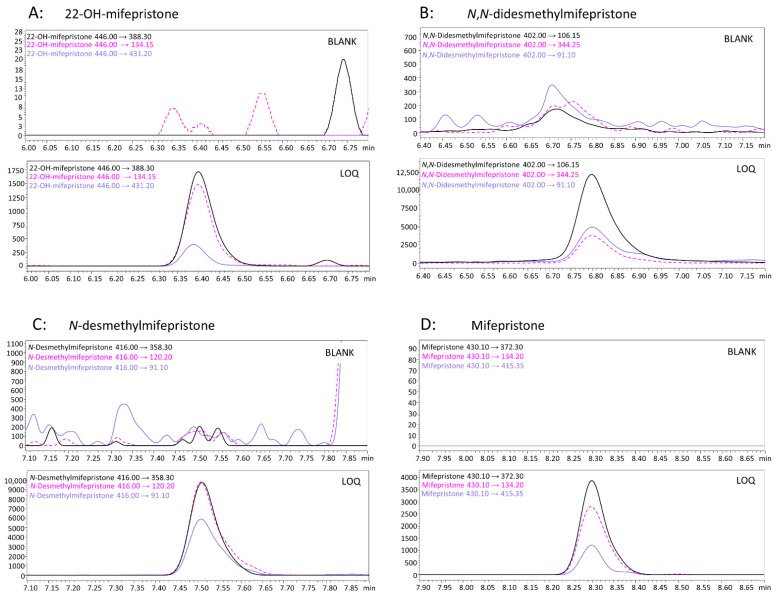
Multiple reaction monitoring (MRM) chromatograms of blank samples and LOQ samples achieved for determined compounds: (**A**) 22-OH-mifepristone; (**B**) *N,N*-didesmethyl-mifepristone; (**C**) *N*-desmethyl-mifepristone; and (**D**) mifepristone.

In the described method, very good validation parameters were achieved. No interfering ion current signals were observed at the retention times of mifepristone and its metabolites as well as deuterated analogues. The LOQ was 0.5 ng/mL for all determined compounds. The linear concentration range was from 0.5 to 1000 ng/mL for *N*,*N*-didesmethyl mifepristone and from 0.5 to 500 ng/mL for other substances. The coefficients of determination (*R*^2^) were >0.999. The results of the recovery, matrix effects, intra-day and inter-day precision and accuracy are presented in Table 3. The RSD% and RE% values did not exceed ± 13.2%. The recovery values were in the range of 96.3–114.7% and the matrix effect values were from −3.0 to 14.7%. The extraction efficiency values were calculated by comparison of the peak areas and the recovery values were calculated on the basis of concentrations. Therefore, it is worth noticing that it is not possible to compare the abovementioned data directly. Carryover was acceptable because the peak area of a zero sample analyzed after an injection of ULOQ did not exceed 19% of LOQ for all substances. Presented values are in an accepted ranges in accordance with the GTFCh (Gesellschaft für Toxikologische und Forensische Chemie ang. German Society of Toxicological and Forensic Chemistry) recommendations. Multiple reaction monitoring (MRM) chromatograms of blank samples and determined compounds at LOQ concentration are presented in Figure 1. Short-term stability studies revealed that mifepristone and its metabolites are stable under tested conditions. The percentage bias values were found to be between 0.75 and 10.90%. The largest changes were observed for *N,N*-didesmethyl-mifepristone. The percentage bias values were in the range of 7.05–10.90% for this substance. For the other compounds, 22-OH-mifepristone, *N*-desmethyl-mifepristone and mifepristone, the abovementioned values were in the ranges of: 1.85–5.17%, 0.75–2.23% and 0.82–5.02%, respectively.

### 3.2. Method Application

The developed and fully validated method for the determination of mifepristone with its metabolites in human blood was successfully applied in our laboratory according to forensic toxicological practice. The UHPLC-QqQ-MS/MS method was applied in authentic casework in an analysis of the blood sample from a woman who used RU-486 for a self-induced abortion. Analysis of the maternal blood sample revealed the presence of five compounds: mifepristone (557.4 ng/mL), *N*-desmethyl-mifepristone (638.7 ng/mL), 22-OH-mifepristone (176.9 ng/mL), *N,N*-didesmethyl-mifepristone (144.5 ng/mL) and *N*-desmethyl-hydroxy-mifepristone (qualitatively). Toxicological analysis revealed an absence of misoprostol acid (the main metabolite of misoprostol) and other drugs in the maternal blood. Misoprostol acid was not determined despite the fact that the woman was supposed to take drugs containing misoprostol in their composition. This is most likely related to the rapid metabolism of misoprostol, which makes it undetectable within a short time after ingestion [17]. Diclofenac, an ingredient that is often present with misoprostol in pills [18], was also not detected. The biological material from the fetus was not provided to our laboratory for toxicological analysis. For this reason, determination of abortifacients was not possible.

Table 4 presents a comparison of LC methods for the determination of mifepristone in biological materials. It can be concluded that the method presented in this paper is characterized by the lowest limit of quantification, except for the method developed by Ishii et al. [15]. However, in the abovementioned paper the validation results of the method were not provided. The authors described only the range of the calibration curve, in which the lowest point was a concentration of 0.25 ng/mL. Among the LC-MS methods developed to date, the technique described in this paper is the only one in which mifepristone-*d_3_* was used as an internal standard for the quantification of mifepristone in biological samples. It is noteworthy that the use of deuterated analogues of substances of interest (including mifepristone metabolites) enabled a very high rate of recovery. Furthermore, the presented UHPLC-QqQ-MS/MS method was applied for whole blood samples, which is definitely a more complex biological matrix than plasma or serum. This fact makes our method suitable for use in forensic toxicological practice, in which whole blood is the most widespread biological fluid collected for testing.

In some countries, self-induced pharmacological abortion is not a crime, but it is illegal. In such cases, forensic laboratories routinely examine the blood of dead infants, miscarried fetuses, and placentas secured during the prosecutor’s investigation. Therefore, the method of mifepristone determination in terms of forensic toxicology should be developed for complex matrices instead of plasma (which is rarely tested). Another important issue is the sample volume needed for analysis performance. The presented method does not require a large amount of the sample, which is especially important during the analysis of fetal blood. During the fetus autopsy, it is not always possible to collect several milliliters of biological fluids. Moreover, there is a need to perform a large amount of further analysis of collected biological material (e.g., of other medicines, recreational drugs, or novel psychoactive substances). Most of the methods for the determination of mifepristone applied to date involved time-consuming multistep extraction with the use of SPE [10,11,12,13,14]. In contrast, the LLE extraction technique described in our paper is fast, simple, inexpensive, and does not require the use of large amounts of organic solvents harmful to the environment. Therefore, it can be successfully applied not only in forensic toxicology laboratories but also in other institutions that appreciate these advantages.

### 3.3. Metabolic Pathway of Mifepristone in Humans

**Figure 2:**
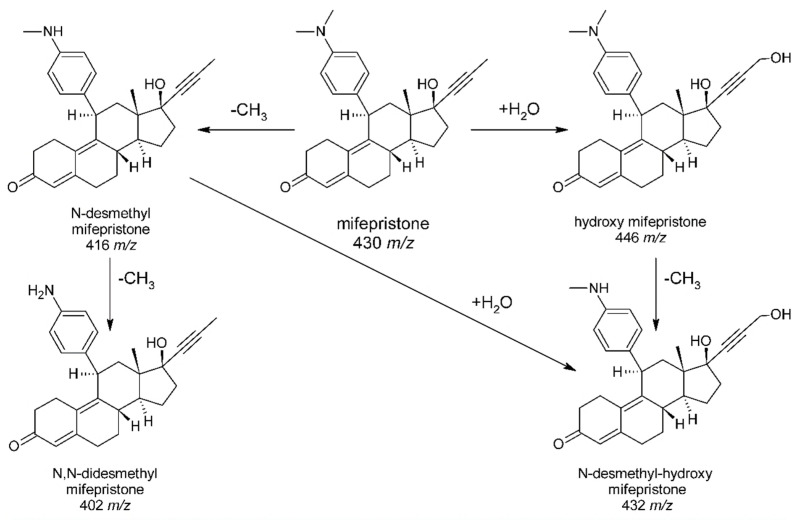
Proposed metabolism of mifepristone (RU-486) in humans.

The identification of mifepristone metabolites in maternal blood sample proves that the applied extraction technique is also suitable for the determination of mifepristone metabolites (whose presence in biological samples is related to biotransformation processes occurring in a liver). In addition, in cases where blood collection was carried out a long time after the use of the drug, the only way to confirm the ingestion of mifepristone may be the identification of its metabolites (especially with a longer half-life than the initial substance). By performing an analysis in the Q3 scan mode, it was possible to identify four metabolites of mifepristone and create a proposed metabolic pathway of RU-486 in humans (presented below in Figure 2), which corresponds to the scheme proposed earlier by Wu et al. [19] in their in vitro studies (human hepatic S9 fractions). Examinations presented in this paper of authentic toxicological casework made it possible to verify and confirm the abovementioned experimental data.

**Figure 3:**
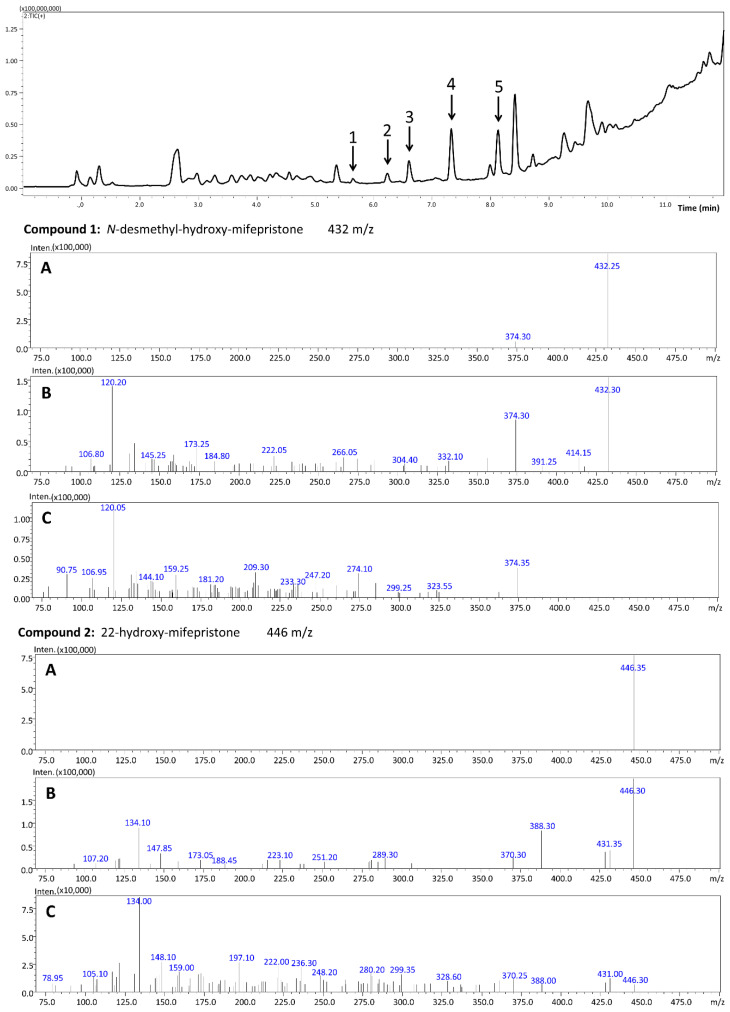
Total ion chromatogram (TIC) of mifepristone and metabolites in blood extract (**top**) and product ion spectra for *N*-desmethyl-hydroxy-mifepristone (precursor ion: 432 *m*/*z*) and 22-hydroxy-mifepristone (precursor ion: 446 *m*/*z*) at three collision energies (**A**) −10 V; (**B**) −20 V and (**C**) −35 V.

**Figure 4:**
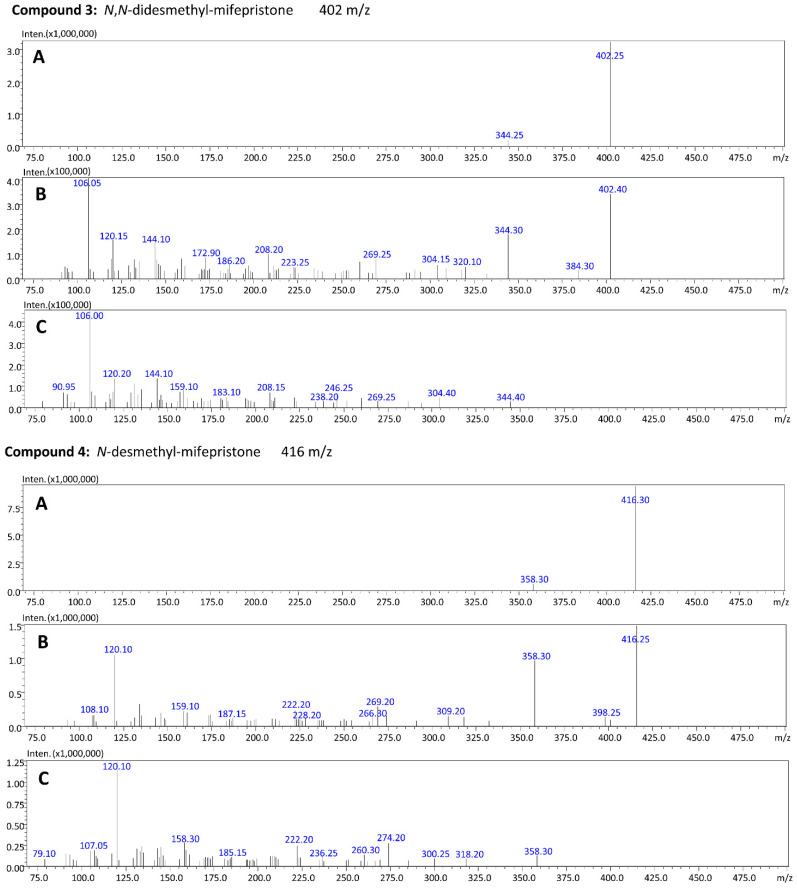
Product ion spectra for *N,N*-didesmethyl-mifepristone (precursor ion: 402 *m*/*z*) and *N*-desmethyl-mifepristone (precursor ion: 416 *m*/*z*) at three collision energies (**A**) −10 V; (**B**) −20 V and (**C**) −35 V.

**Figure 5:**
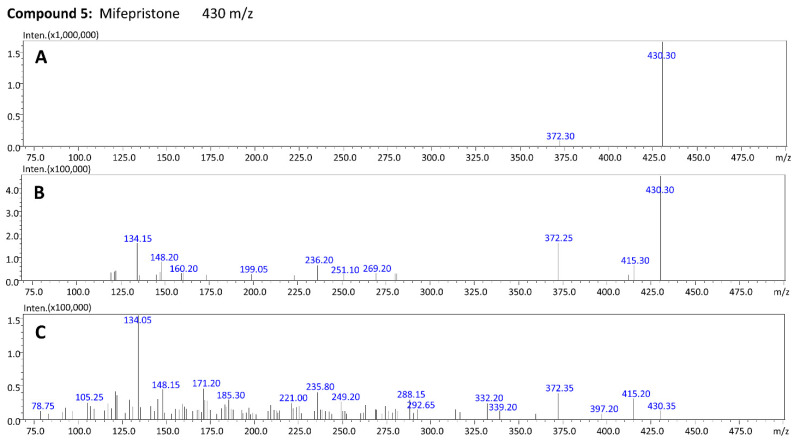
Product ion spectra for mifepristone (precursor ion: 430 *m*/*z*) at three collision energies (**A**) −10 V; (**B**) −20 V and (**C**) −35 V.

The total ion chromatogram (TIC) of the blood extract with mifepristone and its four metabolites is presented in Figure 3. For each compound (marked with an arrow), the product ion scan was performed at three collision energies: −10 V, −20 V and −35 V (Figure 3, Figure 4 and Figure 5). By analyzing the product ion scan spectra, it can be easily observed that various metabolites of mifepristone exhibit differences within the *N,N*-dimethylamine moiety attached to the aromatic ring. The molecular masses of the precursor ions and *m*/*z* ratios of characteristic fragments were the basis of the identification of mifepristone’s metabolites in human blood. Mifepristone and hydroxy-mifepristone both have *N,N*-dimethylamine moiety in their chemical structures (Figure 2). Hydroxyl moiety is attached to the terminal propynyl group. In product ion spectra, a characteristic fragment of 134 *m*/*z* can be observed for the abovementioned compounds (Figure 3 and Figure 5). Ion with a mass to charge ratio of 120 *m*/*z* is formed by the loss of methyl (–14 Da) attached to the nitrogen atom. This fragment is characteristic of *N*-desmethylated compounds: *N*-desmethyl-hydroxy-mifepristone (Figure 3) and *N*-desmethyl-mifepristone (Figure 4). A product ion with a mass to charge ratio of 106 *m*/*z* visible in compound 3 (Figure 4) corresponds to the *N,N*-didesmethylated analogue of mifepristone (134–28 Da). Taking into consideration the precursor ion of compound 1 (432 *m*/*z*) it can be assumed that this metabolite has the hydroxyl group attached to C22; however, the nitrogen atom is desmethylated. Retention times for presented compounds are as follows: *N*-desmethyl-22-hydroxy-mifepristone (5.82 min); 22-hydroxy-mifepristone (6.39 min); *N,N*-didesmethyl-mifepristone (6.80 min); *N*-desmethyl-mifepristone (7.51 min) and mifepristone (8.29 min).

The pharmacokinetics of the mifepristone are characterized by rapid drug absorption and a half-life (t_1/2_) from 25 to 30 h. Interestingly, following ingestion of a single dose of mifepristone (100 to 800 mg), concentrations were all approximately 1000 ng/mL after 24 h in serum samples [20]. In our case, the woman ingested six A-Kare pills, each containing 200 mg of mifepristone and 200 µg of misoprostol (information refers to the original drugs) approximately 24 h before the blood collection. Based on the pharmacokinetic data (maximum blood concentration and a half-life) it seems that the maternal blood concentration (557.4 ng/mL) of mifepristone determined in the presented work corresponds with the previously mentioned values. Information on mifepristone and its metabolites concentration in maternal blood after taking illegal abortifacients pills purchased on the internet is not available. To our knowledge, the blood concentrations of mifepristone and its metabolites in maternal blood samples collected after self-induced abortion have not been reported to date.

The only study in which the mifepristone concentration in biological specimens after an illegal abortion was provided, is the paper by Ishii et al. [15]. Mifepristone’s concentration in the fetal plasma sample was estimated to be 7.1 ng/mL. This concentration is much lower than the concentration determined in the mother’s blood sample (presented casework). It suggests that to prove an illegal abortion with the use of mifepristone, maternal blood is significantly better biological material than fetal blood, because of the higher concentrations of this substance. However, the low concentration of mifepristone in fetal plasma indicates the need for an application of sensitive methods of RU-486 determination in postmortem material. Another noteworthy fact is that pills purchased online may contain different dosages from their legal counterparts [9]. Interest in abortifacient drugs has increased among individuals deciding to carry out pregnancy termination without specialist care. The monitoring of the possibility of the presence of these drugs in biological samples and their quantification are necessary as self-induced abortions are still a current issue.

## 4. Conclusions

A rapid, sensitive, and reliable method for the determination of mifepristone and its metabolites in human blood was developed and fully validated. The presented method was the first one applied to date for the quantification of abovementioned compounds in a maternal blood sample after a self-induced pharmacological abortion. Toxicological analysis revealed the presence of five compounds: mifepristone (557.4 ng/mL), *N*-desmethyl-mifepristone (638.7 ng/mL), 22-OH-mifepristone (176.9 ng/mL), *N,N*-didesmethyl-mifepristone (144.5 ng/mL) and *N*-desmethyl-hydroxy-mifepristone (qualitatively). The established UHPLC-QqQ-MS/MS method is the first applied to date, with the utilization of deuterated analogues of mifepristone’s metabolites, which resulted in very good method parameters such as precision, accuracy and recovery for all determined compounds. The presented method is one of the most sensitive techniques applied so far (0.5 ng/mL) with a simultaneous reduction in the sample volume to only 200 µL. Furthermore, by performing metabolism studies as described in this paper on authentic toxicological casework it was possible to create the proposed metabolism of RU-486 in humans (which eventually confirmed in vitro studies carried out on humans’ hepatocytes). The developed UHPLC-QqQ-MS/MS technique is suitable for the evaluation of the pharmacokinetics, toxicology, bioavailability, and clinical pharmacology of mifepristone in future research as well as in terms of forensic toxicological investigations.

## Figures and Tables

**Table 1:** Multiple reaction monitoring (MRM) conditions used in the UHPLC/ESI-QqQ-MS/MS method for quantification of mifepristone and its metabolites in whole blood samples.

Substance	Precursor Ion [*m*/*z*]	Product Ion [*m*/*z*]	Dwell Time (msec)	Q1 Pre-Bias [V]	Collision Energy [V]	Q3 Pre-Bias [V]
22-OH-mifepristone	446.0	388.3 *	9	−11	−23	−17
		134.15		−11	−30	−24
		431.2		−11	−26	−30
22-OH-mifepristone-*d_6_*	452.0	394.25 *	9	−11	−22	−18
		140.25		−12	−30	−28
		154.3		−10	−28	−30
*N*,*N*-didesmethyl-mifepristone	402.0	106.15 *	9	−10	−31	−10
		344.25		−11	−19	−11
		91.1		−10	−54	−22
*N*-desmethyl-mifepristone	416.0	358.3 *	2	−11	−20	−16
		120.2		−11	−30	−21
		91.1		−10	−55	−19
*N*-desmethyl-mifepristone-*d_3_*	419.0	123.2	2	−11	−27	−24
		361.3 *		−11	−20	−16
		161.2		−11	−34	−15
Mifepristone	430.1	372.3 *	2	−20	−23	−24
		134.2		−20	−31	−22
		415.35		−20	−27	−19
Mifepristone-*d_3_*	433.1	375.3 *	2	−12	−24	−17
		137.2		−12	−33	−21
		415.35		−12	−25	−19

* Ions selected for quantitative analysis.

**Table 2:** Comparison of extraction efficiency [%] obtained for each compound in different pH conditions and with the use of various extraction solvents.

		Extraction Efficiency [%]			
		Mifepristone	*N*-Desmethyl Mifepristone	*N*,*N*-Didesmethyl Mifepristone	22-OH-Mifepristone
pH 7.4	Ethyl acetate	50.3	60.1	46.8	56.1
	*n*-Hexane	20.7	2.9	8.9	0.2
	DCM	29.8	54.8	52.2	47.2
	TBME	55.9	68.5	69.0	89.0
pH 9	Ethyl acetate	49.0	65.6	61.7	78.5
	*n*-Hexane	16.7	2.8	7.8	0.2
	DCM	20.0	36.9	55.9	64.0
	TBME	62.0	71.1	73.0	94.4

Abbreviations: DCM—dichloromethane, TBME—*tert*-butyl-methyl ether.

**Table 3:** Calibration curve and validation parameters of the UHPLC-QqQ-MS/MS method for determination of mifepristone with metabolites in whole blood samples.

Substance	Calibration Curve		Validation Parameters						
	Linear Concentration Range [ng/mL]	Internal Standard	Concentration Level [ng/mL]	Intra-Day		Inter-Day		Recovery [%] *	Matrix Effect [%] *
				Precision [%] *	Accuracy [%] *	Precision [%] *	Accuracy [%] *		
Mifepristone	0.5–500	Mifepristone-*d_3_*	5	2.6	0.1	7.4	−8.4	103.4	3.4
			100	7.8	−5.5	2.6	−2.4	101.1	1.1
			500	5.5	−13.2	6.2	−4.2	97.7	−2.3
*N*-desmethyl mifepristone	0.5–500	*N*-desmethyl mifepristone-*d_3_*	5	2.3	0.5	3.4	1.6	108.0	8.0
			100	9.8	−0.7	9.2	−2.8	97.0	−3.0
			500	2.0	−5.0	9.8	−7.5	110.2	10.2
*N*,*N*-didesmethyl mifepristone	0.5–1000	*N*-desmethyl mifepristone-*d_3_*	5	2.4	3.6	7.6	11.3	103.6	3.6
			100	6.1	−5.3	7.4	−10.4	100.1	0.1
			500	1.1	−8.7	3.3	1.1	114.7	14.7
22-OH-mifepristone	0.5–500	22-OH-mifepristone-*d_6_*	5	0.0	−2.1	11.7	−3.0	96.3	6.3
			100	3.8	−7.2	8.7	−8.8	100.0	0.0
			500	6.5	−12.2	7.2	−4.8	104.8	4.8

* (*n* = 5).

**Table 4:** Comparison of liquid-chromatographic methods applied for mifepristone determination in biological samples.

Matrix (Volume)	Sample Preparation	Method	Recovery/IS	LOQ [ng/mL]	References
Plasma (1000 µL)	SPE (Oasis HLB)	HPLC-UV	95.1–105.8%/norethisterone	10	[10]
Plasma (500 µL)	SPE (Oasis HLB)	HPLC-QqQ-MS/MS	94.5–103.7%/levonorgestrel	5	[14]
Serum (1000 µL)	SPE (Oasis HLB)	HPLC-UV	92.7–104.3%/ mifepristone analogue	10	[11]
Plasma (1000 µL)	SPE (Oasis HLB)	HPLC-UV	91.7–100.1%/norethisterone	10	[12]
Plasma (100 µL)	Protein precipitation with ACN	UPLC-QqQ-MS/MS	–/alfaxalone	0.25–the lowest calibration level	[15]
Plasma (500 µL)	SPE (Oasis HLB)	HPLC-UV	94.7–101.2%/–	20	[13]
Whole blood (200 µL)	LLE (pH 9; TBME)	UHPLC-QqQ-MS/MS	97.7–103.4%/mifepristone-*d_3_*	0.5	Presented method *

Abbreviations: SPE—solid-phase extraction; ACN—acetonitrile; LLE—liquid-liquid extraction; TBME—*tert*-butyl-methyl ether; HPLC—high-performance liquid chromatography; UV—ultra-visible detector; QqQ—triple quadrupole; MS/MS—tandem mass spectrometry; IS—internal standard; LOQ—limit of quantification; * method allowing for simultaneous quantification of mifepristone’s metabolites; – information was not provided.

## Data Availability

Not applicable.

## Sample Availability

Samples of the compounds are available from the authors.
